# To What Extent Is Typhoid Fever Preventable?

**Published:** 1897-06

**Authors:** J. L. Hildreth

**Affiliations:** Cambridge, Mass.


### TO WHAT EXTENT IS TYPHOID FEVER PREVENTABLE?[1]

BY J. L. HILDRETH, M.D., CAMBRIDGE, MASS.

Not long ago I read something that interested me very much with reference to the practice of law in London, in which a distinguished judge said that if one of his predecessors of a hundred years ago was to come back and sit upon the bench, he would find

[1] Read before the American Academy of Dental Science, January 6, 1897.

it just as easy as when he sat there before. I thought to myself that we were rather proud that we could not make any such boast about medicine. The man who would attempt to practise medicine this year as he practised it twenty-five years ago would certainly not maintain his hold upon the community, nor do as good work as those around him, and he would be looking for work elsewhere. The contrast is very forcibly brought out in this comparison between medicine and law; the practice of medicine is progressive, advancing from day to day, while the law probably remains as it did one hundred years ago.

I suppose it would be fair to say that this progress, which medicine has made, has been no more signal in any direction, and no more important in its results than in regard to our knowledge of the cause and the successful treatment of diphtheria. It certainly stands foremost in the work which the profession has done for humanity. Next to that, I think we may place typhoid fever. We cannot take the same forward step with regard to the treatment of typhoid fever as with diphtheria, and prescribe and use the antitoxin, but I think it is fair to say, with reference to typhoid fever, that we know quite as much about the cause and the course of the disease, the way in which it gets its hold upon the system, and the effect which it has on the patient,—we know a great deal more about it than we do with reference to diphtheria; and yet, when your committee asked what should be put on the card, I purposely put it in the form of a question, because I do not know that I, or any other medical man, could answer the question as to how far typhoid fever is a preventable disease.

I simply thought that I would state to you the facts with reference to typhoid fever as they are known to the profession to-day. Of course, I cannot expect that these facts will interest you as they do us who are in the harness of every-day work and seeing it everywhere, but they have great interest to the layman, as I think I may demonstrate to you, and after I have stated these facts, then I will let you, each one for himself, answer the question if he may, as to how far it is a preventable disease.

I know that in every-day life things that are very familiar, that are all about us, that are common with us, do not impress us as those things that we but rarely see. I remember, in the last epidemic of small-pox, that Boston was crazy over thirty or forty cases, while the fact that one-seventh of all the deaths that occurred in the city was caused by consumption awakened no thought or interest among the people, although the number of deaths was a hundred-fold more from consumption than from small-pox. There is that same lack of interest when we speak of a middle-aged person having typhoid fever, because we see so much of it and have seen so much of it all our lives, but when you consider the great amount of typhoid fever that occurs every autumn, from August to November, and when you consider the length of sickness of each individual case, if the case runs a successful course, together with the term of disability which follows after the disappearance of the disease, and when you consider the amount that has been expended in the payment of the bill for nursing and the doctor's bill for a single case, and multiply that by the total number of cases, you will be surprised at the enormous amount which typhoid fever alone costs the people of Massachusetts. Take, for instance, our little Cambridge Hospital, which has had an unusually large number of cases the past six months, July to January, and where we have but thirty-four beds; we have entered eighty cases, having about fifty there at one time which we were obliged to do our best to care for on account of the great number that demanded admission. Now, if you stop to think that each case costs the hospital for the care and treatment (making no account of the cost of the plant, which originally cost something like one hundred thousand dollars, besides the equipment) about thirteen dollars; then if you remember that most of these cases that get into the hospital will certainly average as much as six weeks; then multiply six by thirteen and that by eighty, and you will see that you have a sum a little above six thousand dollars, which it must have cost our Cambridge Hospital this fall for typhoid fever alone. The cost to those persons who are able to pay for their treatment is no trifling amount, the expense of a nurse for three or four weeks and the attendance of a physician and the other incidental expenses. I think your president can well remember his first year in Cambridge when he had typhoid fever, and that the expense of it was quite a large sum. You know that a person is not only disabled in earning capacity during the continuance of the disease, but, many times, for two or three months afterwards; so that the expense of typhoid fever in Massachusetts, if we knew how much it cost to take care of the sick ones, and added to that the loss of wages,—which means a great deal to poor families, and even to those who are well-to-do,—would seem almost incredible. This is not all. The death-rate during most epidemics is rarely less than ten per cent., and oftentimes comes up to twelve, thirteen, and even fifteen per cent., so that the distress is great in addition to the loss.

There have been some very interesting epidemics of typhoid fever, and I will refer to the more remarkable, some of the earlier ones which attracted attention and which started the investigations which brought us to where we are now in our knowledge with reference to the development and extension of the disease. The investigation of one of these epidemics showed the facts to be that a man was going home from Vermont, and passed the night in a little village near the eastern border of New York. He was then suffering from the beginning of an attack of typhoid fever, the earlier symptoms of weakness, diarrhœa, etc., and through his diarrhœal discharges he contaminated a well which was used in the village where he passed the night, and in that village, the population of which numbered nine families, a total of forty-three persons, one-half of them contracted the fever and ten of them died. You will remember, the most of you, the great number of sick people who came from Philadelphia at the time of the Centennial in 1876, and were found to have typhoid fever. Nobody will ever know how many cases or deaths from typhoid fever could be traced to that exposition, or what was the cause of the epidemic, although it is very probable that the drinking-water was contaminated. A great many of these epidemics have been traced to the contamination of drinking-water, and the English people always speak of such epidemics as "water-borne" typhoid. One of the most interesting of these water-borne epidemics occurred in 1877 along the banks of the Ohio River. The water in the river became contaminated with the excreta from some case or cases, and the whole length of the river, a distance of some eight hundred miles, it was one epidemic of typhoid fever. Then in the town of Plymouth, Pennsylvania, in the year 1886, there was a contamination of a water-supply which was used by about eight thousand of the inhabitants. There were one thousand cases of typhoid fever in that town at about the same time, of which one hundred died, a mortality of ten per cent., and of those who contracted the fever, seventy-five or eighty cases were taken sick on precisely the same day.

You know, of course, that the number of cases of typhoid which were contracted at Chicago, during the World's Fair, was very large, but it was undoubtedly very much lessened by the precaution which they took to introduce Waukesha water, and a very few people, comparatively, used the water of the lake. I suppose if you should go into the larger cities, you would find a great many people who came home sick from the fever, contracted in Chicago,—I know that is certainly true of Cambridge,—but the number was not so large as it certainly would have been if they had not taken the precautions they did.

With reference to armies, death by typhoid fever often reduces the ranks as much as the enemies' bullets. In the Civil War the number who died from typhoid was enormous. I tried once to get the statistics, but they wrote me from Washington that they had never been published. The statistics of the French army, for the period from 1875 to 1891, estimate the number of cases of typhoid fever to have been one hundred and thirty thousand, of which ten to fifteen per cent. died. You know that we had in Lowell a few years ago an epidemic which was caused by the excreta from three persons contaminating the water there, the Merrimac, at Chelmsford. I was asked to go there and see some of the cases; there was a difference of opinion as to whether they were really typhoid or not, but when I went into one of the wards and saw fifteen or sixteen cases in a room it reminded me of the hospital tents during the war-times. At about the same time thirty or forty cases appeared in Lawrence, and an investigation showed that the epidemic along the river was undoubtedly due to the contamination of the water by the excreta from those three original cases.

These epidemics of typhoid fever from contaminated water-supply have been known not only here, but have also been recognized in England. One of the most notable outbreaks was that which was known as the "Blue Hill epidemic," in which some of the excreta from two cases was cast out on the side of a hill in the winter time, and there came a rain and a melting of the snow, and this excreta was taken into the water and carried down to a town thirty or forty miles below, where the water was used for drinking purposes, and a very large number of people contracted the disease.

Later investigations have proved that typhoid can be conveyed by the use of milk, so that we now have milk epidemics as well as water-borne ones. One of the earliest of these milk epidemics took place in Cambridge, and for the successful investigation and the undoubted proof of its origin the Board of Health in Massachusetts has great praise. Since that time their expert, Professor Sedgwick, has examined twenty-eight epidemics which he has traced to the contamination of milk. The large number of cases which we had in Cambridge the past year was almost entirely due to milk. We have had three milk epidemics there in one year. I trust this may not influence any one from going there to live and making use of the educational advantages which we have to bestow, and appreciating the beauty and convenience of the city as a place of residence,—but we have been unfortunate this year. In one of the epidemics seventy cases came down in ten days, and of these, sixty of them occurred in the route of one man. The proprietor of the milk-route was a very fine man, and he had no idea that the milk which was being delivered to his customers was carrying sickness and death to so many of them.

Another source of conveying the disease is oysters. As we sat at dinner I conversed with my friend from Providence, and I thought of the Middletown epidemic which they were unable to account for until they found that a great many of those who had been taken with the fever had eaten oysters which were grown in a portion of the river close to which there had previously been a couple of cases of typhoid fever, and it was learned that the dejectures were thrown into the river. English people have done more than we have in working up oyster epidemics. Fortunately, they are rare, but oysters must not be overlooked in the investigation of an epidemic.

Now, these sources of conveying the disease to patients are the ways that we know most about, but I fancy, as time goes on, many other ways by which the disease is conveyed will become known. But we have been convinced of one thing: that the germs of typhoid fever enter the body through the intestinal track, and probably in no other way. Our investigations have led us to the belief that no exhalations from any dirty source, no offensive smells or putrefying substances, will cause typhoid fever; that the drinking-water may be bad, vile in every way, smell, taste, and appearance, but unless it has present in it the germ of the disease, the typhoid bacillus, it will not produce typhoid fever. Now, this is the result of the later studies and knowledge that have been acquired in reference to this disease. We know that oysters and milk and water convey typhoid fever, but we do not know that it may not be conveyed in other ways. If you should take the statistics of a hospital and go over the typhoid fever cases and see, if it were possible, how many were water and milk cases you would find a great number which must have been contracted from some other source. Now, if you stop to think, you will see that those things that I have alluded to—milk, water, and oysters—are about the only food which we take into the system that is uncooked,—aside from fruit, as for instance, the banana, the apple, and the orange. These are not usually cooked, and it is possible may be sources of conveying typhoid fever and other diseases as well. Also, there is another reason, which is that milk and water are believed to be the media in which the typhoid germs grow with the greatest rapidity; they are the best media for their culture, except some artificial media which are made from bouillon or something of that sort. Pettenkofer, who was really an extraordinary man, and who spent a great deal of time in the investigation of fevers, and Louis, another man who took a deep interest in the study of fevers, believed in the theory that typhoid fever originated from the ground in some way and that it might float in the atmosphere, and thus cause epidemics at certain times and places. We know of nothing to prove that emanation from the ground or from any unclean or decaying substance will produce typhoid fever, although I do not see why the germ should not occasionally get into the intestinal tracts through the air-passages.

Gerhard, of Philadelphia, in 1834, demonstrated that typhoid fever and typhus fever were distinct diseases. Before that time they were regarded as the same disease, although they knew that some cases presented ulcers in the intestines, while others did not, and these they called abdominal typhus. On the other side, Sir William Jenner, who gave us so much work on vaccination, published, in 1849, a valuable paper, in which he stated that the two diseases were then conceded to be quite different. Typhoid fever is due to lesions in the intestines and digestive organs, and in Peyer's patches, and the glands which are carrying on the process of digestion. Typhus fever is essentially a disease of the blood. We do not know very much about the cause of typhus fever,—that is, what germ is present in the disease,—but we know that it seems to find a very desirable habitation in jails and houses where men and women are crowded, and where they give little attention to cleanliness. It was this disease which was present in almost all of the ships of England in Howard's time, and was looked upon as a dreadful plague, as it certainly was.

The bacillus which is present in typhoid fever is very much like another bacillus which is present in the intestinal tract in health, not only in man, but in many animals. It is present in the cow and the horse, but, I believe, it is not present in the dog. It is called the *coli communis.* That bacillus when viewed under the microscope without any particular stain seems very much like the bacillus of typhoid, but it grows in a different media, and looks quite different when it is subjected to certain stains. Under the microscope these bacilli may be seen very readily with a twelfth immersion, but not with a power much below that. These photographs that I show you are simply to show the different shape and form and different lengths which they manifest when they are presented under the microscope. They are copied from a photograph by Sternberg, who has done a great deal of work in bacteriology, and are quite true to nature. The ordinary bacillus will average perhaps a twentieth of an inch in length when it is magnified one thousand diameters. As I have told you, the bacillus of typhoid looks very much like the *coli communis* which is present in nearly all animals, and is the commonest thing in the intestinal tract. Just here let me say something which may interest you and which is of great interest in the investigation of typhoid epidemics. Where you are looking for contaminated drinking-water, the present method is not simply by making chemical tests, not by taking a specimen of the water and placing it in a cone-shaped filter and examining the sediment under the microscope, but by making cultures from this sediment and growing them in various kinds of culture media, and staining them until the bacilli can be completely identified. I have no doubt that a great many mistakes were made by those who, years ago in examining drinking-water, found this bacillus which they thought the typhoid, but which was only the *coli communis.* It is not unlikely that many times the water examined contained the excreta from some horse or cow or other animal, and not understanding the difficulty of separating the *coli communis* from the typhoid bacillus, no doubt many mistakes were made,—that is to say, you cannot take the bacillus and stain it with one stain and put it under the microscope and say that it is the typhoid bacillus; you must go through quite a process of staining before you can be sure of the identity of the bacillus.

The discovery of this germ (although the possibility of its existence had been suspected by quite a number of workers) was made by Eberth, who discovered it and announced it in 1888, so it is less than ten years that we have known positively that there was a typhoid bacillus, and that in no case does the disease exist without it.

This is where we stand at present in our knowledge of this disease,—that the typhoid bacillus *must* be present; that it probably enters the body in no other way than through the intestinal tract, and there finds a medium which is agreeable and conducive to its propagation, and in some way gets into the glands which we call Peyer's patches, which secrete the material used in digestion, and also in the spleen. The spleen is a particularly favorable place for their growth, and it is said that you can get almost a pure culture of the typhoid bacillus from the spleen of a person who has died with typhoid. Now, while these parts are the most favorable for the reproduction of the bacillus, and are what may be called its home, yet if it occasionally gets into the circulation,—by that I mean into the blood,—and is carried about and locates itself in other parts of the body, then we have what is called a typhoid septicæmia, a general poisoning of the body. These cases of typhoid take on very much the same form, and the person affected has much the same temperature-chart as one who is suffering from galloping consumption or miliary tuberculosis. Now, this is about all that we actually know with reference to the typhoid bacillus. We know nothing of its life outside of the body, or where it lives, or what it does. A good many laboratory experiments have been made with reference to its life in water, and it has been known to live for weeks and sometimes for months in water about the temperature of the body. Pruden froze the typhoid bacillus in a block of ice and kept it there for months, and when it was thawed out, he found the bacilli as lively and active and ready for work as if fresh from a patient. It is a very lively bacillus, always moving, kicking, and working about in all directions. No one has ever been able to take a bacillus and cultivate it and see it with anything below the twelfth immersion under the microscope, and even then it moves so rapidly in every direction that it is almost impossible to tell what it looks like. Of course, if you stain it you destroy its activity.

It is probable that this bacillus has a life outside of the body where it lives and propagates, and that we simply see it in its wanderings when it gets into the human body and causes so much trouble. What is still more remarkable is the fact that it is not the bacillus itself that does the harm. Its behavior and its life in the human body are precisely the same as with diphtheria bacillus, except that it does not produce its effect so quickly. The bacillus of diphtheria, although it is such a dreadful thing, does not of itself produce much trouble. It is the toxin which it generates and which is absorbed into the system that causes all the trouble. And the same is true of the typhoid bacillus: it is the toxin which it produces that causes the disease we call typhoid.

Now, right here I might say that it differs from diphtheria in one respect, and it is because of this difference that our hope of the future is very much less than with diphtheria. As I have before stated, the bacillus of diphtheria produces a toxin, and it is that toxin that causes the sickness and death of the patient.

Now, we have learned that as soon as that toxin is present in the system of the patient nature at once goes to work to manufacture an antitoxin which counteracts the effect of the toxin, and the patient's life is saved in that way. It has also been discovered, and I may say that it is one of the most important discoveries ever made by the medical profession, that we can produce diphtheria in the horse, and as the system of the horse manufactures the antitoxin, we may extract the horse's blood, and by injecting the serum into the diphtheritic patients assist them in overcoming the effect of the toxin and making the system an uncongenial habitation for the bacillus.

Unfortunately, typhoid fever cannot be produced in any of the lower animals. Very many experiments have been made in trying to produce the disease by inoculating horses, dogs, cats, rabbits, but without successful results. If we could produce typhoid fever in the lower animals, then we would go to work and extract the serum which they have made to enable them to throw off the disease and inject it into typhoid patients and thereby hasten the immunizing process which goes on in each individual, and so save many lives, just as we have been able to do in the case of diphtheria; but no animal has yet been found that can have the typhoid fever, and so far we have no hope of making an antitoxin which we can inject into the bodies of persons suffering from typhoid fever, and by serum therapy assist them in overcoming the disease.

It is a very curious fact with reference to the bacilli of diphtheria that while we understand more about them, we do not know how soon after being taken into the mouth they begin to manufacture their toxin, but we know that they work very rapidly, and begin to make their presence felt perhaps within a day. In typhoid fever, so many observations have been made that we know almost to a certainty that a person who drinks water which is contaminated by the typhoid germ—if they are susceptible to the disease—will be taken sick by the fourteenth day, so that, in case of a milk epidemic, for instance, if a person has taken contaminated milk, and passes by the sixteenth, or seventeenth, or eighteenth day, you may feel pretty sure he will not come down with the disease.

Before the introduction of the antitoxin treatment for diphtheria we used to say, when we were doing our best to combat the bacillus of diphtheria, that if the patient passed the fourth day he stood some chance; if he passed the sixth day, he stood a greater chance, and after the eighth day he was pretty sure to get well. With regard to typhoid fever, we now say that the twenty-first day usually marks the crisis. I have been looking over the records of some of the cases in our hospitals, and I have been surprised to see how closely this rule is followed. In most of the cases the patient began to get normal after the twenty-first day, unless there was a relapse, a return of the disease, showing that the system had not manufactured antitoxin enough to control the disease. I had a good opportunity to observe this regularity in the time of the last epidemic in Cambridge and the epidemic in Somerville a few years ago. In tracing the route of the milkman who supplied the milk, it was learned that every one of the patients came down on the same day, almost precisely,—I think there were thirty-three cases that came down within one or two days. In that epidemic—the Somerville one—the young man who had the distribution of the milk, the milkman's son, had typhoid fever, and no doubt he was uncleanly in his habits and some of the dejections from the bowels got into the milk, which was a beautiful place for the germs to live, and consequently the disease was communicated to those who drank the milk. The Cambridge epidemic was due to a young fellow who had what we call "walking typhoid,"—that is, while he felt sick, he continued to work through it all, not being sick enough to require him to leave his work. When these outbreaks occurred, you could take a map and, by following the routes of these milkmen, mark down the limits of the epidemic. Of course, not all the people on these routes were taken down, because it is rarely, after forty, that a person contracts the disease, and, besides, some persons are not susceptible to the disease, just as you know some persons are not susceptible to small-pox or any of the ordinary contagious diseases.

There has been an attempt made to treat typhoid fever in the same way that Pasteur undertook to treat hydrophobia, which, as you know, was by taking the essence of the disease, the spinal cord, and making cultures from it, and injecting that, in dilute solutions at first and gradually increasing the quantities, immunizing them more and more until they became so that they were not susceptible to the disease. That was his method of treating rabies, and to some extent he thought he was successful. A method something similar to this was tried in typhoid fever last year, in the hope of cutting short the disease, with some good results; but it did not commend itself to the profession, and it has not been carried on this year.

It was early known that typhoid fever was contracted a great deal in the country, and that it was caused by contaminated wells; and even before the presence of the germ was fully understood, the physicians in Boston made prophecies that if the water-supply of Boston was free from contamination, typhoid fever would disappear from that city. Dr. Henry I. Bowditch made that prophecy many years ago. In cities and towns where they are very particular with reference to their water-supply, and especially in those places where great care is also taken with regard to the use of milk, as they do in many of the cities on the other side, typhoid fever has been very nearly driven out. In Berlin and Munich, where there were severe epidemics some fifteen years ago, typhoid fever has disappeared now to such an extent, and is so rare, that the teachers in the medical schools and at the hospitals are scarcely able to find cases enough to teach their students the characteristics and appearances of the disease. I will mention here that the Germans rarely use raw milk; they will tell you that it is not fit to use, and I presume it is for that reason, and the efficient care that is taken of their water-supplies, that cases are not plenty enough in those cities for the teaching of the clinical appearance and effects of typhoid fever. Within a month or two one of the most distinguished physicians of Baltimore stated before a medical meeting that if the water-supply and drainage of Baltimore was put where it should be and the milk that was used was sterilized, he would guarantee the disappearance of typhoid fever from that city within three years. Many of our prominent physicians believe that if we were to use no milk except that which is cooked or sterilized, typhoid fever would soon be as rare with us as small-pox.

Now, you see that this brings a very important public matter to our minds; if these things are true,—and I believe them to be true,—you can see the great responsibility that rests upon the boards of health and the people who have charge of our water-supplies, and also upon the boards of health who have charge of our milk-supplies, to see that the water and milk which is supplied to our citizens is free from contamination, if you stop to consider for a moment the possible effects of this contamination. Take, for instance, the water-supply of Boston: if there should occur a case or two of typhoid fever on the borders of one of the basins, and some of these germs should get into the water of that basin, the bacillus, being such a lively creature and having such vitality, would soon find his way through the water-pipes and the faucets to those who used the water. It is very fortunate those things have not occurred; and this is another reason why boards of health are so strenuous and determined in requiring that all cases of typhoid fever shall be reported to them. Fortunately, also, we have material, like corrosive sublimate and carbolic acid, which does destroy this bacillus. One of the most deadly mixtures which we can use for this purpose is the common lime-water that is used to whitewash fences. This is used at the hospitals, to be mixed with the excreta. Beside this, the most extreme care is taken that the persons who handle any of these dejections, or any of the clothing about the patient, shall wash his hands so that he may not convey the disease to others.

Boards of health of the cities and towns carry on various investigations and inspections still further. The State Board began quite a number of years ago to employ Professor Sedgwick in the investigation of these epidemics of typhoid fever. He goes to work in a very methodical manner: visiting the houses where there are cases of typhoid, he sits down and asks all about each case; finds out where they get their milk; asks if this patient drank the milk, and whether other members of the family use the same milk; and with these facts which he obtains he is usually able to locate the origin of the epidemic. It is a very delicate and painstaking task to work the problem out, but you would be surprised to see how satisfactory the results are. Oftentimes you would say, "That case is not a milk case," and you would look for another cause. In my investigation of one of the Somerville cases this summer, when I asked the question, "Did you get the milk of such a milkman?" they said "No, but of Mr. ——." As there was no epidemic on his route, I said to myself, "Now, this must be one of those cases which must have been contracted in a way other than by the use of contaminated milk, and it will be extremely difficult to locate its origin;" but the question was settled when the woman remembered and said, "But that man does buy milk from the other man when he is short of milk himself." Another difficulty you have to contend with is the assertion by the milkman that no one connected with the handling of the milk has had typhoid fever. One of the Somerville epidemics illustrates this point: Mr. —— did not believe that his son who handled the milk was suffering from typhoid fever; but when the young man died, we were able to show by the autopsy that he had had typhoid fever. Another instance of the difficulty of getting the true facts is well illustrated by a case that occurred at an institution where they raise their own milk. They had three or four cases, the origin of which puzzled the doctors very much. They produced their own milk, and as they had no cases at their institution, they did not see how it was possible for any one there to contract the disease. It turned out, afterwards, that when they were short of milk, they occasionally bought milk from the milkman whose son had died from the fever, and that this milk was used only by those sitting at one table, and that all of the cases came from among those who used this milk, and that none of those who used the milk they produced had the fever. You see how much can be done in working out the origin of these cases if sufficient time is taken, and you also see the reasons for the belief that many epidemics of typhoid fever are caused by contaminated milk. It seems to me that, to all intents and purposes, the proof is conclusive; at any rate, it is so accepted by medical men.

As I said in the early part of my remarks regarding water, that it might be filthy, vile, contaminated with all sorts of things, yet it would not produce the fever in those who drank it unless it contained the germs of typhoid fever. And so it is with milk Milk may produce diarrhœa, may produce vomiting, might be contaminated with various bad things, but the use of it does not produce typhoid fever unless the germ of typhoid fever is present in the milk. This discovery we consider one of the triumphs of medicine,—this knowledge that typhoid fever is dependent upon this germ alone and nothing else. How and to what extent it is a preventable disease, you see, it is not easy to answer. I believe, and I think that almost all the medical men believe, that in all probability fully nine-tenths of all the cases of typhoid fever are caused either by water or milk that is contaminated with typhoid germs. If we should adopt the custom that the Germans have, and use no milk unless it has been cooked, I believe we would get rid of a very large proportion of the cases we have each year. It is not necessary that the milk be brought to the boiling-point to make it perfectly safe; it may be Pasteurized,—that is, brought to a heat of 60° or 70° C., and kept at that point for some fifteen or twenty minutes. If treated in this way, it is rather more palatable, as well as digestible, than if it has been brought to the boiling-point.

At the present time boards of health are doing all they can, and doctors are doing all they can, to find when there are epidemics of this fever; and now that the people understand this matter better, when there is an epidemic, they will either stop using milk altogether or have it boiled; and, besides this, if there is a suspicion regarding the water, they will boil that also.

With these facts that I have given you, I will leave you to answer for yourselves how far typhoid fever is a preventable disease. And I will also ask you why we cannot achieve as much in this line of preventive medicine as has been achieved in Munich and Berlin within the last fifteen years in lessening the large number of cases of typhoid fever that every autumn fill our hospitals and bring so much distress and suffering to patients, as well as to their friends who have to care for them and minister unto them?

